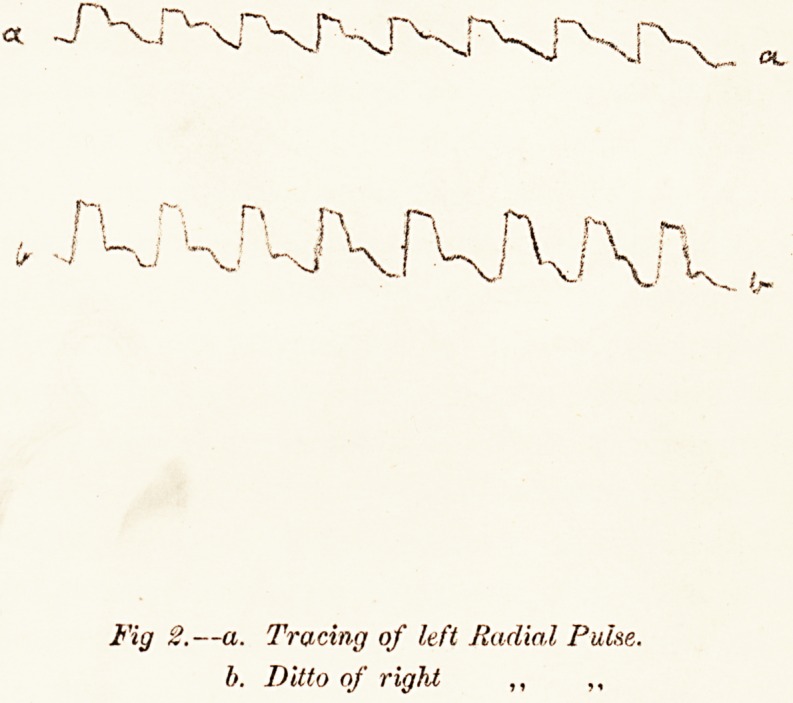# Case of Aneurism of Aorta—Treatment by Galvano-Punctures: Result Encouraging

**Published:** 1886-12

**Authors:** R. Shingleton Smith

**Affiliations:** Physician to the Bristol Royal Infirmary


### CASE OF ANEURISM OF AORTA—TREATMENT BY GALVANO-PUNCTURES: RESULT ENCOURAGING. By R. SHINGLETON SMITH, M.D., F.R.C.P., Physician to the Bristol Royal Infirmary.

Susan S., æt. 29; married ten years; two children—one of them still-born, the other died at seven months; one miscarriage. No definite history of syphilis, beyond falling of hair and occasional sore throat. Had typhoid fever ten years previously, and rheumatic fever fourteen years ago. No history of intemperance.

Was first admitted to Bristol Royal Infirmary on November 4th, 1882, complaining of pain in right side of chest, dyspnœa, dysphagia, and other symptoms suggestive of aneurism. The patient dated her symptoms from October, 1881; when, lifting something heavy, she felt as if "something suddenly snapped in her chest," and severe pain obliged her to desist from her occupation and lie down. A few days afterwards the pain diminished; but it had never since left her entirely. It had varied much in intensity and position, but became more and more constant. Her breathing became difficult, and there was some dysphagia. On admission, the pain was through the right side of chest, from the sternum to the shoulder, and down the right arm. It was increased on swallowing, and was so severe as to require hypodermic injections of morphia.

There was dulness at the right infra-clavicular region, extending to the fourth intercostal space; also at the supra-spinous and infra-spinous regions of the same side, as low as the middle of the scapula.

There was a slight systolic bruit in the second right interspace, and some little displacement of the heart's apex; but no definite tumour or abnormal pulsation could be made out.

The patient was put on Tufnell's diet, and treated with iodide of potassium, in doses increasing from 10 to 60 grains, three times daily.

January, 1883. There was little change. Occasional attacks of pain were present; but no increase of the symptoms.

June, 1883. The dysphagia had entirely passed off, and the pain had much diminished.

On July 19th she was made an out-patient, and then took ordinary diet.

October 23rd. Was re-admitted; and again made out-patient on January 29th, 1884.

October 28th, 1884. Patient had continued to attend as out-patient, and had taken the iodide of potassium in reduced doses. The symptoms had varied in intensity from time to time; but she had never been altogether free from pain: had often experienced difficulty in swallowing, and occasional spasmodic cough, with some bronchitis. About a week ago the pain had increased in severity, and she was again made in-patient.

At this time she had a careworn and anxious appearance, with eyes somewhat prominent, and veins on right side of neck distended. There was no evidence of defective nutrition, and the axillary temperature was normal on both sides. The left radial pulse was a little stronger than right. There was now distinct bulging at the upper part of the chest; and a diffused pulsation could be felt on the right of the sternum, at the second and third interspaces. The sternum was distinctly prominent—most so at the level of the second interspace. No bruit could be heard over any part of the swelling. There was some displacement of the apex beat, and a systolic apex murmur.

The voice was a little husky, and there was frequent dry cough. Loud tracheal breathing was audible at the second right space near to the sternum, and behind at the right interscapular region, where there was also some percussion dulness. Patient was again put on a limited minimum diet, and iodide of potassium in doses of 40 grains.

November 24th, 1884. There was no material improvement, and the patient complained much of pain and dyspnœa: accordingly, it was resolved to employ the galvanic needles. No anæsthetic was given by inhalation, but cocaine was used locally. One needle was inserted at the second right interspace, and was kept in for twenty-five minutes; the negative pole being connected with a large sponge, which was held to the surface and varied in position. Patient did not complain much of pain at the time, but a few hours afterwards required a hypodermic injection of morphia. The day following, it was thought that the swelling had increased slightly, and the cough was very troublesome.

A second operation was performed on November 30th, when two needles were inserted, and retained for thirty-two minutes, with a current at first from twenty-five Leclanché cells, afterwards from twenty, and for the last ten minutes from only fifteen cells. No anæsthetic was used, locally or generally. Both needles were attached to the positive pole, and the negative pole was applied by means of a sponge to the surface of the tumour. The passage of the galvanic current appeared to give rise to considerable pain at the sites of puncture; so much so, that the current was reduced in intensity, in order that it might be continued. This second operation was followed by distinct improvement: two days after, the pain had much abated, there was less dyspnœa, also less cough; and the patient believed that the swelling "did not heave so much." Ten days afterwards the improvement had continued, and the pulsation had distinctly diminished.

January 10th, 1885. There had been more pain in the arm and shoulder; and the patient, encouraged by the result of the previous operations, wished to continue the galvanic treatment. Accordingly, two needles were again inserted, cocaine having been previously injected at the site of each puncture. The needles gave rise to much pain whilst the current was passing; but it was continued, more or less steadily, for eighty minutes. Ten days later there was a little improvement, but no decided change in the condition of the swelling. The punctures had on each occasion healed without difficulty, but a little black blood had usually escaped when the needle was withdrawn.

January 31st. The sternum was decidedly more prominent than before; there had been more dyspnœa, and some duskiness of face. Patient was again anxious to have the needles, as she was satisfied they had previously given much relief. A fourth operation was performed on February 14th, two needles being introduced, one on either side of sternum, in the second intercostal space, and retained for one hour, with a current of twenty-five cells. No anæsthetic was used. The expansile pulsation of the sac was well demonstrated by the movement of the two needles, one on either side. On the following day the patient was very comfortable, and the heaving pulsation was less marked; but the skin at the punctures appeared to be inflamed to an area of a quarter of an inch in diameter. The tracheal breath-sound, both in front and behind, was less marked. A fortnight later, March 1st, there was a definite improvement: the heaving impulse was slowly diminishing, and the prominence of sternum less marked; the pains were less frequent, and the breathing fairly easy. For about a month there was no indication for further interference; but, on consultation, it was agreed that more active galvanic treatment was justifiable, in the hope that it would do more than alleviate distressing symptoms. Accordingly, on May 13th, a fifth operation of a more energetic character was performed. It was intended that a larger number of insulated needles, all attached to the positive pole, should be passed into the sac; and this was scarcely possible without anæsthetic. On previous occasions it had been found that ether by inhalation gave rise to much excitement and dangerous struggling, and therefore anæsthesia was induced by means of chloroform. Four needles were introduced to a depth of two inches, two on the right and two on the left of the sternum; the bone, although softened, prominent, and pulsating, afforded a barrier which the needle would not pierce. A current from thirty cells was maintained for half an hour, the negative pole being in contact with the skin by means of a sponge rheophore, the position of which around the tumour was frequently varied. On removal of the needles, no bleeding took place from those on the right; but there was copious oozing of black fluid blood from those on the left, which was at last arrested by collodion pads and pressure. During this operation the patient was profoundly unconscious and cyanosed; the breathing had been much distressed, and the chloroform had been discontinued after the first five minutes, because of cyanosis and other indications of asphyxia. The pulse continued regular, but small; and artificial respiration maintained the action of the heart, in spite of very deep cyanosis and venous engorgement. Unconsciousness continued from 12.40 to 3 p.m., when the breathing power returned, and artificial aid was discontinued. After return of consciousness there was no evidence of paralysis, or any indication of embolism. At 5 p.m., there being some complaint of pain, a small morphia draught was given, and the patient slept quietly for five hours.

On the following day the patient was of good colour, the cyanosis having disappeared; the breathing was easy; there was no dysphagia, or cough; and no pulsation could be detected by the hand on the tumour. Pulse 116, regular.

Two days later there was some cough, with purulent expectoration, and stridulous breathing, with a little return of cyanosis: on the day after, epistaxis, with laboured breathing, and decided pulsation in tumour.

One month after the fifth and the major operation, there had been very marked and steady improvement: there was no dyspnœa, no cyanosis; pulsation was less decided; and the aneurism felt firmer, as if more consolidated, although the area of dulness remained stationary.

On July 31st she was able to get up and move about. The tumour was not increasing, and there were no active symptoms. Accordingly, she was allowed to go home, and present herself from time to time as an out-patient. This she continued to do through the winter; but on April 26th, 1886, she was re-admitted in consequence of a very violent attack of general bronchial catarrh, which commenced four days before. There was much dyspnœa and cyanosis, with cough and expectoration; but no œdema of legs, no hæmoptysis, and no increase of the aneurismal signs. The air entered both lungs equally, and the cough was not of spasmodic character; but there were loose râles on both sides. There was no decided pulsation in the aneurism; but the prominence and area of dulness were as before.

On the following day there was copious expectoration by the aid of an apomorphia mixture (gr. $\frac{1}{24}$) with hydrobromic acid and ether; but on the day after, the patient expired, four hours after an injection of one-third of a grain of morphia.

On examination after death, it was found that the upper portion of the sternum was pushed forward by a large aortic aneurism, adherent to the periosteum behind; the bone was very thin, as also were the coats of the sac. The aneurism involved the first and second parts of the arch; was as large as a child's head at birth; was soft and yielding, but pressing equally on the trachea, just above its bifurcation; on the bronchi, more especially the right, and the œsophagus; but neither tube was materially occluded or eroded.

The lungs were full of frothy mucus; there was much bronchial congestion, with œdema and partial consolidation at both bases.

On the visceral layer of the pericardium covering the aorta was a large patch of old inflammatory lymph (probably the result of the operative interference).

The aneurismal sac was opened from the front: it was firmly adherent to the sternum, which was sawn through longitudinally, and was nowhere entirely perforated. The cavity contained much soft *post-mortem* clot; but a laminated mass of old firm fibrine (one inch and a half thick) occupied the anterior portion of the sac, was adherent to the wall, and may have been the result of the numerous operations. No laminated clot existed in any other part of the sac; and here it was exactly what one might expect to occur from the induction of coagulation in foci, by means of galvanic action. The walls of the sac were thin, but composed of all the arterial coats. The aneurism was sacculated; it grew from the right front wall of the aorta; its orifice was about three inches in diameter and seven in circumference. A secondary thin-walled sac bulged from the posterior wall of the main cavity. The lumen of the great vessels springing from the arch was not obstructed; nor were the veins compressed.

No marks of the galvano-punctures could be detected in the walls of the sac.

This case, watched continuously throughout a protracted course of nearly four years, and diagnosed from the onset as one of aneurism of aorta, derives its principal interest from the question of treatment. Iodide of potassium, in combination with rest and a limited diet, appeared to arrest the course of the disease: the symptoms abated, and the patient considered herself well enough to leave the hospital ward, and go to her own home, after nine months' treatment.

On being re-admitted three months after, the symptoms were again relieved; and she was discharged, fairly well, in a little over three months.

Nine months later, galvanic treatment was commenced: first with one needle introduced into the sac; then, a week later, with two needles, insulated except at the ends; five weeks later two needles; and after another interval of four weeks, two needles for the third time. There was

*Fig 1.—*
*a. Outer wall of Aneurismal Sac.*
*b. Interior of Sac.*
*c. Left Carotid Artery.*
*d. Left Subclavian Artery.*
*e. Part of Sternum sawn through.*
*f. Interior of Aorta.*
*g. Laminated clot lining the Aneurism.*
*(N.B.—This has not been made thick enough in the Drawing).*
*h. Wall of Sac (cut edge).*

*Fig 2.—a. Tracing of left Radial Pulse.*
*b. Ditto of right ,, ,,*

then an interval of three months; but the patient would willingly have had earlier and more frequent operations, as she was convinced of the relief given by the previous ones. The fifth operation, apart from the question of anæsthesia, gave a very encouraging result, and certainly supports the view that galvano-puncture, in spite of much adverse criticism and experience, when carried out in a particular manner, is likely to be of benefit, even in so formidable a disease. Even now text-books advise that the needles should be connected with both poles of the battery; whereas it is well-known that firm coagulation takes place on the positive pole only, and gas is evolved at the negative needles, which must materially interfere with the formation of a firm clot inside the sac. There can, therefore, be no advantage in having the negative pole introduced through the skin; and, on the other hand, it must be an advantage to have a considerable number of needles, all connected with the positive pole, which may act as so many centres of coagulation, the clot around the various centres forming a large coherent mass, if the needles be sufficiently numerous and near to each other. In this case, the condition of peril of the patient soon after the commencement of anæsthetic, prevented the introduction of more than four needles at one time, and these must have been at a considerable distance from each other; nevertheless, the condition found after death appears to indicate that the various operations had induced some amount of coagulation in the part of the sac which the needles had reached. From the clinical point of view the benefit was decided; and the fact that the patient repeatedly wished and asked for further operative interference, is the best possible evidence of the relief afforded to symptoms from time to time.

After the last operation, the general health continued to be better than before for about twelve months; the question of further interference having been avoided, in consequence of the danger of anæsthesia, without which no such operation as is likely to be efficient would be practicable. Death took place at last from a severe attack of double catarrhal broncho-pneumonia—no doubt influenced by and aggravated by the aneurism; but, manifestly, not set up by or due to the aneurism itself.

It cannot be claimed that the aneurism was cured by galvano-puncture: its progress was, however, arrested; and the death of the patient arose from another disease, nearly five years after the first commencement of the symptoms, and nearly four years after the presence of aneurism of the arch had been recognised.

This case does, therefore, give encouragement to the further trial of a method of treatment which has hitherto been somewhat disappointing, but which may yet be productive of far more decided and more permanent benefit than has as yet been observed.

---

## Figures and Tables

**Fig 1. f1:**
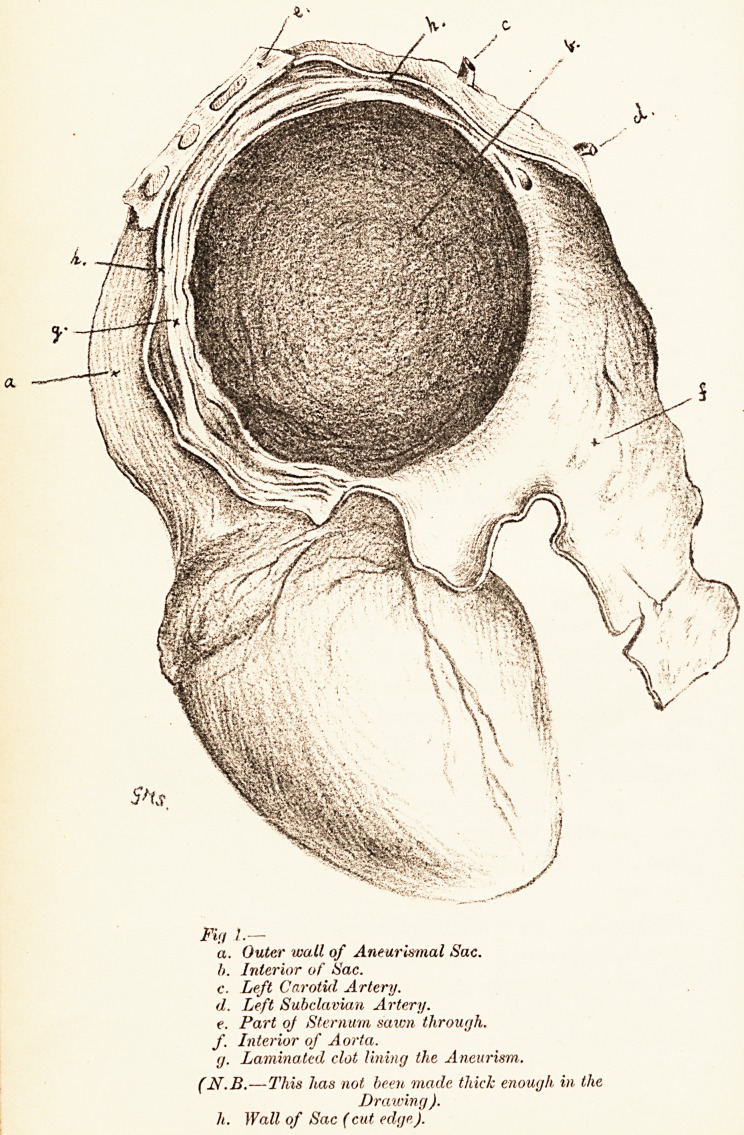


**Fig 2. f2:**